# LoRaWAN for Vehicular Networking: Field Tests for Vehicle-to-Roadside Communication

**DOI:** 10.3390/s24061801

**Published:** 2024-03-11

**Authors:** Gabriele Di Renzone, Stefano Parrino, Giacomo Peruzzi, Alessandro Pozzebon, Lorenzo Vangelista

**Affiliations:** 1 Department of Information Engineering, University of Pisa, 56122 Pisa, Italy; gabriele.direnzone@phd.unipi.it; 2 Department of Information Engineering and Mathematics, University of Siena, 53100 Siena, Italy; parrino2@unisi.it; 3 Department of Information Engineering, University of Padova, 35131 Padova, Italy; giacomo.peruzzi@unipd.it (G.P.); lorenzo.vangelista@unipd.it (L.V.)

**Keywords:** IoT, LoRaWAN, transmission performance analysis, wireless links in motion, vehicular wireless networks

## Abstract

Vehicular wireless networks are one of the most valuable tools for monitoring platforms in the automotive domain. At the same time, Internet of Things (IoT) solutions are playing a crucial role in the same framework, allowing users to connect to vehicles in order to gather data related to their working cycle. Such tasks can be accomplished by resorting to either cellular or non-cellular wireless technologies. While the former can ensure low latency but require high running costs, the latter can be employed in quasi-real-time applications but definitely reduce costs. To this end, this paper proposes the results of two measurement campaigns aimed at assessing the performance of the long-range wide-area network (LoRaWAN) protocol when it is exploited as an enabling technology to provide vehicles with connectivity. Performances are evaluated in terms of packet loss (PL) and received signal strength indicator (RSSI) in wireless links. The two testing scenarios consisted of a transmitter installed on a motorbike running on an elliptical track and a receiver placed in the centre of the track, and a transmitter installed on the roof of a car and a receiver placed next to a straight road. Several speeds were tested, and all the spreading factors (SFs) foreseen by the protocol were examined, showing that the Doppler effect has a marginal influence on the receiving performance of the technology, and that, on the whole, performance is not significantly affected by the speed. Such results prove the feasibility of LoRaWAN links for vehicular network purposes.

## 1. Introduction

Vehicular networks represent a paradigm in which the fundamentals of mobile wireless networks are applied to the field of vehicles, meaning that they represent the nodes of wireless sensor networks (WSNs) that are capable of transmitting sundry information (e.g., the state of the vehicles, environmental variables of the cabins, parameters related to loaded goods, etc.). In addition, and thanks to the advent of the Internet of Things (IoT), vehicular networks have evolved into the broader framework of the Internet of Vehicles (IoV), stressing the paradigm in which vehicles equipped with sensors and actuators are given Internet connectivity to fulfil, for instance, remote monitoring and control. More generally, vehicular networks include the architectures of vehicle-to-vehicle (V2V) and vehicle-to-roadside (V2R) communications models; the latter is under investigation in this paper.

Concerning the enabling technologies, they can be distinguished into two broad categories: cellular and non-cellular. The former, like long-term evolution (LTE) or 5G, are nearly mandatory whenever ultra-reliable low-latency communications (URLLC) or massive IoV applications are needed. However, using cellular technologies implicitly implies a notable increment of running costs since data transmission plans must be subscribed to. On the other hand, employing non-cellular technologies, like long range (LoRa) modulation and long-range wide-area network (LoRaWAN) protocol, results in a lower quality of service (QoS), thus limiting the range of application scenarios but at the same time drastically cutting the running costs. Therefore, a trade-off has to be usually reached in the design phase of vehicular network infrastructures.

Thanks to their reliability and robustness, LoRa modulation and the LoRaWAN protocol have been widely exploited in a plethora of distributed monitoring applications, thus becoming an almost de facto standard low-power wide-area network (LPWAN) technology within the IoT framework. Such a result is achieved because of the fact that LoRa is grounded on the chirp spread spectrum (CSS) modulation, which has the capability of demodulating signals to which a notable level of noise was added during transmission. Indeed, LoRa modulation can fully operate below the noise floor level, meaning that signals that have very low signal-to-noise ratio (SNR) (e.g., in the order of magnitude of −20 dB) can be demodulated. This also translates into a maximum receiver sensitivity of −137 dBm, depending on the exploited transmission parameters like the spreading factor (SF), the bandwidth, and the coding rate (CR). Such a feature resulted in the adoption of LoRaWAN in a myriad of application scenarios, and especially in the harsher ones: in non-line-of-sight (NLOS) conditions [1,2], in industrial contexts [3,4], in underground applications [5,6], in marine and offshore environments [7,8], and in search and rescue procedures in remote areas [9,10].

This paper extends a previous work [11], in which the behaviour of LoRa modulation and LoRaWAN protocol was investigated in cases of vehicles in motion. Specifically, a transmitter was installed onboard a motorcycle, and while it was transmitting data, the vehicle was driven along an elliptical track at several testing speeds. At the same time, a LoRaWAN gateway was placed at the centre of the track, and the packet loss (PL) and received signal strength indicators (RSSIs) were measured. Such results are reported herein to be compared with the ones related to a new measurement campaign in which the LoRaWAN transmitter was installed on the roof of a car. Similarly, the transmitter sent data while the car was driven on a straight road at several velocities, and the gateway was placed next to the road at the halfway point. This testing setup allowed us to assess the performance of the transmitting technology from the point of view not only of the PL and RSSI, like the former experiments, but also of the Doppler effect. Indeed, this phenomenon was hardly assessable in the former campaign since the distance between the transmitter and the receiver remained almost constant throughout the tests. Therefore, by means of the results related to the field measurement campaigns, this paper contributes a deeper assessment of the capabilities of the LoRaWAN protocol in enabling V2R networks by analysing metrics related to the receiver side (i.e., PL and RSSI) and by investigating whether the Doppler effect affects such wireless links and, if so, evaluating to what extent. In particular, these results, which at first glimpse appear to be contradictory with respect to the expected theoretical behaviour, allow us to fully understand and demonstrate the actual behaviour and performances of LoRaWAN receivers in terms of frequency tolerance, showing the possibilities that they offer within the field of vehicular networks. Moreover, another goal of this work is to preliminary validate the feasibility of V2R links exploiting LoRa modulation and the LoRaWAN protocol (i.e., two technologies that were not designed and developed for this particular application scenario).

The rest of the paper is drawn up as follows. Section 2 presents related works about the topic, highlights their similarities and discrepancies with this work, and clarifies how this paper advances the current state-of-the-art. Section 3 briefly explains the Doppler effect, while Section 4 presents the experimental setup from the point of view of the hardware and the instruments that were exploited for the field tests, which are shown in Section 5. Then, Section 6 is devoted to proposing the obtained results, their comparison, and their discussion. Finally, Section 8 highlights conclusions and final remarks.

## 2. Related Works

Vehicular networks can be enabled by the most diverse wireless communication technologies. Of course, the requirements of the application scenario at hand prescribe the most suitable transmission protocol. For instance, if low latency is mandatory, then 5G is by far the most appropriate. On the other hand, if low power consumption and low running costs are at a premium, then LPWAN technologies (e.g., LoRaWAN) are the most suitable choice. However, provided that a plethora of wireless transmitting technologies can do the job, only related works dealing with LoRa modulation and LoRaWAN protocol are herein treated because this work is focused on such solutions. In doing so, a complete and fair comparison can be made, since, for example, from the mere perspective of receiving performances, a communication technology operating in licensed frequency bands (e.g., 5G) generally outperforms another standards operating in industrial, scientific, and medical (ISM) frequency bands (e.g., LoRaWAN).

Providing vehicles with wireless connectivity may be useful in order to perform remote monitoring of their state, achieving onboard diagnostics in a quasi-real-time fashion when LoRaWAN is exploited for data transmission, provided that enough gateways are densely installed, for instance, in a smart city paradigm [12]. Similarly, connected vehicles may be critical in emergency or rescue situations. For instance, road accidents can be prevented by setting up V2V communications: vehicles can autonomously recognise whether other vehicles are present in their blind spot and eventually signal to them in such an event by means of LoRaWAN links [13]. Moreover, in cases in which car crashes occur, vehicles can automatically detect such events and consequently call for rescue and emergency personnel, aiming to avoid casualties [14].

Within the smart cities’ domain, vehicular networks play a key role too, for instance, in public transport and shared mobility. Considering a bike-sharing system, although it applies to any other vehicles, one of the most compelling problems to address is the tracking of the vehicles in order to counteract vandalism and thefts. Each bike can be given a Global Positioning System (GPS) module and a wireless transceiver that sends its geographic coordinates on a fixed-time basis. In order to reduce running costs for data transmission, the LoRaWAN protocol can be exploited instead of cellular technologies, as was shown and tested in [15]. Similarly, public transport vehicles (e.g., buses [16,17]) can be tracked by employing a similar infrastructure. However, the localisation of mobile assets like vehicles can be accomplished not only by resorting to GPS but also by analysing fingerprinting of multiple RSSI measurements, provided that multiple gateways are installed [18].

So far, related work has been shown to deal with application use cases. However, the literature also proposes the contribution of analysing the transmission performance of LoRaWAN-enabled vehicular networks, which can be assessed by means of both simulations and field tests, thus more closely resembling the topic of this paper. For instance, in [19], the LoRaWAN protocol was evaluated by means of simulations, accounting for three different sets of transmission parameters (two values of SF and bandwidth were tested) over six different testing setups (simulating several road types and several velocities), and accounting for bit error rate (BER) and SNR as metrics. This study highlighted that transmissions that have a longer symbol time are characterised by worse performance due to the Doppler effect, since, especially at high speed, the coherence time related to the Doppler effect is shorter than the symbol time. However, no field tests were performed. On the other hand, in [20] field tests were accomplished by testing LoRaWAN links between electric vehicle charging stations, which act as gateways, and moving electric vehicles, which act as transmitters, within a smart city environment in Pamplona, Spain. The tests accounted for several NLOS links (due to buildings, trees, cars, etc.) covering the maximum distance of 350m and proving the feasibility of the communication technology from both the point of view of RSSI and SNR measurements. However, no extensive experiments varying the vehicles speed and the adopted SF were performed. Contrarily, in [21], field tests were performed evaluating the effectiveness of LoRa links for vehicular networks in a medium-sized city. Such tests assessed the PL and RSSI when the number of transmitters (installed onboard as many vehicles), the covered distance, the speed of the vehicles, and the transmission parameters (i.e., SF, CR, and bandwidth) varied. The obtained results showed that the performance of LoRa modulation is affected when the distance and the number of transmitters are scaled up for a given SF, CR, and bandwidth. At the same time, when SF = 9, CR = 4/5, and a bandwidth of 250kHz is selected, then the best RSSIs were recorded on average. Although such a result is valuable when IoV infrastructures are designed, it should be noted that the LoRaWAN protocol allows for the exploitation of a 125kHz bandwidth for uplinks (at least in Europe), and this is the reason why we did not test other values for such a parameter. Also, [22] performed field tests setting up an architecture in which a LoRaWAN transmitter was installed onboard a car and several gateways were placed on the road. Concerning the PL, it was found out that it proportionally increased with the car speed, still proving the feasibility of wireless technology for such an application scenario.

Simulations aimed at studying the LoRa channel for vehicular networks, as well as field tests, were accomplished in [23], whose results highlighted that exploiting lower SFs helps in making LoRa links more robust towards the Doppler effect. This is reasonable since symbol time becomes shorter for lower SFs, and at a given speed, the coherence time will be more likely to be longer than the symbol time. This condition became more evident in V2V links whenever the two endpoints were in motion. However, [23] only tested the minimum and the maximum SF, thus providing results for the two extrema but missing a proper analysis for all the SFs in between, as was performed in this paper. On the other hand, in [24] only simulations were performed and accounted for a workspace of 1km^2^ and 2500 moving nodes at a speed up to 90km/h. The relative results showed that performance, in terms of PL, became worse when the number of transmitters increased, as well as when the speed and the payload length increased. But, on the whole, the LoRaWAN protocol proved to be suitable for the application scenario, but no field tests were performed. Similarly, by resorting to simulations, [25] devised an algorithm implementing an alternative adaptive data rate (ADR) scheme to be employed in LoRaWAN-enabled vehicular networks, whose aim was twofold: enhancing performance and reducing power consumption by limiting retransmissions in case of communication breakdown. But, once again, no field tests were performed. Similarly, [26] proposed an alternative version of ADR, pursuing the same goals and obtaining similar results. Another contribution supporting the thesis that better performance can be achieved with lower SFs is [27], where simulations and field tests were performed by testing mobile gateways rather than transmitters. Once again, the authors’ claims were supported by the theory standing behind the Doppler effect and, especially, the relation between the coherence time as varying with speed and the symbol time as primarily varying with the SF for a given payload length. The same authors continued investigating the problem in [28] by setting up several measurement campaigns; one of those resembled one in this paper. Indeed, from this point of view, [28] turned out to be the most similar related work with respect to this paper, although in [28], gateways played the role of moving agents while we exploited moving transmitters. But, at least theoretically, this can be considered as a minor diversity. From the perspective of the obtained results, similarities arose. Despite the viability of a LoRaWAN infrastructure for vehicular networks being fully proven, a performance degradation occurred for high speeds and high SFs. From this, although it is a niche application scenario, even boats were shown to form vehicular networks enabled by LoRa modulation, as in [29] for fluvial contexts. Such a study proved the feasibility of LoRa links in these frameworks, where a transmitter was installed onboard a boat sailing on a river and several gateways were placed on the banks of the river. The relative results showed that an average PL of 22% was experienced. However, apart from the fact that such a measurement campaign was structured in a different way with respect to the one in this paper, the application scenario of [29] is harsh in itself since wireless links are taking place above a water basin, thus introducing an additional variable hindering the wireless channel, as was also proven in a previous work [30].

The literature proposes a plethora of work dealing with LoRaWAN-enabled vehicular networks. The bulk of it is focused more on the application scenario and the monitoring infrastructure as the big picture than on the analysis of transmission performance. Some contributions belonging to the former case were described above to provide readers with a broader perspective on the topic. On the other hand, fewer studies belonging to the latter case can be retrieved, and the most valuable and significant were introduced above. Although the results obtained by such related works are all meaningful and beneficial, to the best of our knowledge, we found a lack in the state-of-the-art concerning a comparative performance analysis like the one we are presenting, especially tackling two use cases (i.e., vehicles travelling on tracks and vehicles travelling on roads) that are of typical interest in real-world applications whenever V2R networks are needed.

## 3. Doppler Shift

LoRa modulation directly derives from CSS modulation, entailing high sensitivity at the receiver side as well as robustness towards multipath interference and fading. However, as the relative transceivers may traverse varying environments at given velocities, the Doppler effect occurs, potentially influencing the performance of LoRa links. Such a phenomenon is observed whenever there is relative motion between a signal source and an observer, where, in this case, they respectively translate into a transmitter and a receiver. In the context of LoRa communication, this effect manifests as a frequency shift in the received signal due to the motion of either the transmitter or the receiver. Understanding the impact of the Doppler effect is pivotal to ensuring reliable LoRa links in all contexts in which devices are in motion (e.g., vehicular networks, asset tracking, mobile sensor networks, etc.). This section delves into the intricacies of the Doppler effect occurring for LoRa links by examining how frequency shifts take place and affect signal quality, thus shedding light on the challenges and potential solutions for maintaining reliable connectivity where mobile transceivers are concerned. LoRa packets are composed of modulated chirps (i.e., symbols) with a given SF quantifying the number of bits per symbol over a given bandwidth BW. This implicitly implies that SF and BW do affect the symbol duration $t_{s}$ as

(1) $$t_{s}=\frac{BW}{2^{SF}}.$$

Generally, whenever a transmitter and a receiver move while they are communicating, the Doppler effect comes into play. In particular, and without losing generality, let us consider a moving LoRa transmitter at speed $v_{tx}$ and a LoRa receiver (i.e., the gateway) standing still. In addition, if the gateway is not on the transmitter trajectory, then the gateway observes the transmitter moving at its radial velocity $v_{rad}=v_{tx}cos\left(\theta \right)$, where θ is the angle between the transmitter’s forward velocity (i.e., $v_{tx}$) and the line of sight from the transmitter to the gateway. Thus, if the transmitter sends a signal with a carrier frequency $f_{0}$, then the receiver detects the frequency *f* according to

(2) $$f=\frac{c}{c\pm v_{rad}}f_{0},$$

where $v_{rad}$ is subtracted when the transmitter becomes closer to the gateway, while it is summed otherwise.

Alternatively, this phenomenon can be described by means of the frequency shift Δf (i.e., the Doppler shift)

(3) $$\Delta f=f-f_{0}=\left[\frac{c}{c\pm v_{rad}}-1\right]f_{0}.$$

Moreover, in a digital communication system (e.g., LoRaWAN networks), the channel response may change over time. However, a timespan during which the channel impulse response can be considered to be invariant can be defined. It is named as coherence time, $T_{C}$, and it is related to Doppler shift since it is its dual in the time domain, in other words,

(4) $$T_{C}=\frac{1}{\Delta f}.$$

Therefore, in order to avoid distortion at the gateway side as a result of the amplitude and phase changes entailed by the alteration of the channel response that occurs due to the Doppler effect, the symbol duration $t_{s}$ should be smaller than the coherence time $T_{C}$. We are going to comment on this in Section 5.2.

## 4. Experimental Setup

Both measurement campaigns share the same experimental setup from the point of view of the transmitter and the receiver. The transmitter included a LoRa transceiver (i.e., the RFM95 from HopeRF) driven by a microcontroller (i.e., the ATtiny84-A from Microchip). Such components were powered exploiting a Panasonic NCR18650B battery proving 3.7V and 3400mAh. However, two different antennas were adopted due to constraints related to the vehicles involved in the two testing scenarios. The tests performed in a velodrome and on a straight road were carried out using, respectively, a motorcycle and a car. The reasons motivating this choice will be provided in the next section. For the tests carried out in the velodrome, a 2dBi λ/8 omnidirectional whip antenna was exploited, while for the tests on the straight road, a 2dBi λ/2 omnidirectional whip antenna was adopted. Despite being different sizes, they provided the same gain, thus reducing the number of involved variables. Unfortunately, using the same transmitting antenna for both measurement campaigns was unfeasible because the λ/8 antenna was placed inside the plastic tail trunk of the motorcycle, which was not big enough to contain the λ/2 antenna. On the other hand, exploiting the λ/8 antenna from the car cabin was not an optimal solution since, contrary to the plastic tail trunk, the car chassis inevitably acts as a source of loss (which cannot be ascribed to movement) within the wireless path loss. Instead, the λ/2 antenna had a permanent magnet at its bottom that was exploited to attach the antenna to the car roof.

The receiver was a LoRaWAN gateway, and included a LoRaWAN concentrator (i.e., the RAK831 from RAKWireless) driven by a Raspberry Pi 3 model B. The concentrator was connected to a 10dBi omnidirectional antenna, while the gateway was mains-powered via an inverter drawing power from a 12V 80Ah lead acid battery.

Several speeds were tested in both scenarios, and for each velocity, many LoRaWAN packets were broadcast by exploiting the following transmission parameters: 6 SFs ranging from 7 to 12, CR of 4/5, bandwidth of 125kHz, payload of 10B, and transmitter power output of 14dBm. Such a configuration was selected to reproduce a worst-case scenario; indeed, lower CRs theoretically improve the ability to correctly restore data at the receiver side. Concerning the number of transmitted packets, two approaches were followed. In the velodrome, 200packets were sent for each speed and for each SF. Conversely, on the straight road, it was not feasible to fix a predetermined number of packets since the time that the car spent travelling on the straight road decreased as the speed increased, meaning that the higher the speed, the fewer the transmittable packets on the straight road. In addition, owing to the frequency hopping scheme established by the LoRaWAN protocol, 8 different channels belonging to the 863–870 MHz ISM band (i.e., 867.1MHz, 867.3MHz, 867.5MHz, 867.7MHz, 867.9MHz, 868.1MHz, 868.3MHz, and 868.5MHz) were exploited for the transmissions in both measurement campaigns.

The gateway was in charge of receiving, demodulating, and forwarding the incoming packet towards a remote network server by making use of the message queue telemetry transport (MQTT) protocol. To this end, the gateway was provided with a 4G dongle, proving Internet connectivity. Moreover, upon receiving, the gateway also measured the RSSIs associated with each of the correctly demodulated packets.

## 5. Field Tests

As anticipated, LoRaWAN links’ feasibility in motion was validated in a velodrome to test cases in which the transmitter orbits around the gateway and on a straight road to analyse cases in which the transmitter passes by the gateway. As was previously stated, a motorbike was exploited in the velodrome, while a car was used on the straight road. Although employing a car would have been optimal because its cruise control could have been exploited to maintain a stable speed, the shape and radius of the velodrome would have significantly limited the maximum reachable speed. On the other hand, this constraint was not present for the tests on the straight road.

### 5.1. Velodrome Measurement Campaign

**Figure 1:**
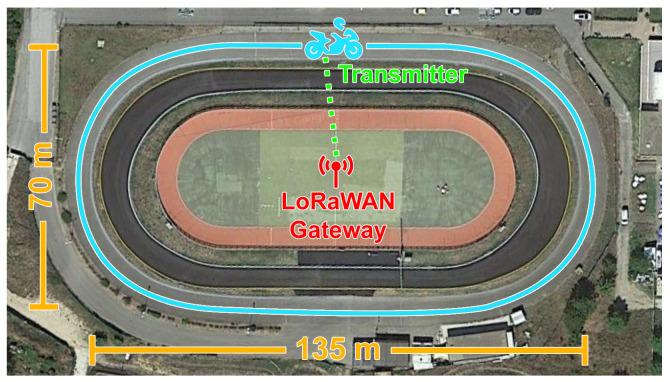
Aerial view of the elliptical track on which the velodrome measurement campaign was conducted (picture taken from [11]).

The velodrome is an elliptical track located in Siena, Italy, whose axes have dimensions of 135m and 70m (see Figure 1). The gateway was placed in the centre of the ellipse in order to limit the variation in the distance between it and the transceiver, consequently reducing the effect of path loss. Moreover, since the transmitter and gateway antennas were omnidirectional and because the transmitter revolved around the gateway, maintaining an almost constant distance, the Doppler effect could be considered negligible. The motorbike was driven along the track at different velocities while the transmitter was broadcasting packets sweeping SFs from 7 to 12 for each of the tested speeds. This measurement campaign accounted for six test sets, during which the motorbike was kept at constant velocities (i.e., 20km/h, 30km/h, 40km/h, 50km/h, and 60km/h) for the former five, while during the last set (marked as ’Max’), the motorbike was driven at a variable speed spanning 60km/h to 90km/h. Keeping a constant speed greater than 60km/h for the entire track was not feasible for safety reasons. In so doing, the effect of movement on both RSSI and PL can be assessed in all those cases in which the distance separating the transmitter and the gateway remains almost constant over time by analysing speed and SF.

### 5.2. Straight Road Measurement Campaign

**Figure 2:**
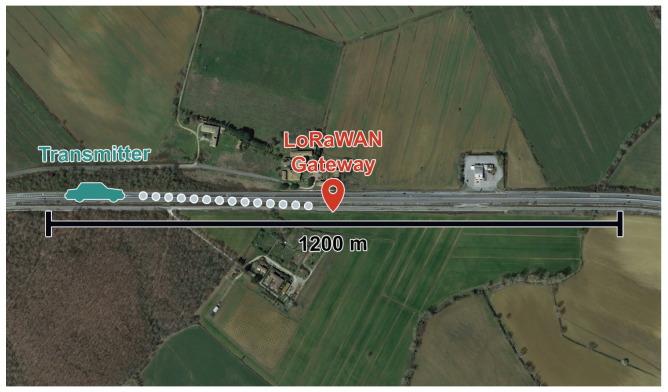
Aerial view of the road on which the straight road measurement campaign was conducted.

The straight road test site is located near Siena, Italy, in the locality of Le Corneta. It is a 1200m long straight road on which no traffic was experienced during the tests since it is a secondary road (see Figure 2). The gateway was placed halfway (i.e., at 600m from both of the endpoints of the road) in a lateral position at 6m from the road. In such settings, the Doppler effect can be perceived on the gateway side. Indeed, this measurement campaign had a twofold scope by accounting for several speeds and all of the SFs: on the one hand, we assessed the Doppler effect on PL considering both the leg in which the transmitter approaches the gateway, and the one in which it leaves the gateway; on the other hand, we assessed the effect of movement on RSSI whenever the distance separating the transmitter and the gateway varied over time.

Tests were carried out by travelling the straight road several times in the same direction. The car was driven at 50km/h, 70km/h, 90km/h, and 110km/h by setting its cruise control. Each speed was tested twice by travelling the straight road for as long as all of the SFs were tested. Prior to the tests involving movement, some RSSI measurements were taken by placing the transmitter at fixed points in order to assess whether movement can significantly affect RSSI. Such spots were at the starting point and, in turn, at 300m, 600m, 900m, and 1200m from the starting point (therefore, the last spot corresponded with the finishing point of the road), and at each location, 100 packets per SF were broadcast by the transmitter.

#### Doppler Effect Analysis for the Straight Road Measurement Campaign

As discussed in Section 5.1, the Doppler effect can be considered negligible in the case of the velodrome. Conversely, it has to be taken into account in the case of the straight road. Therefore, in order to forecast the expected behaviour of LoRa technology in this context and the expected capability of correctly receiving packets in the presence of the Doppler effect for different radio settings, the relation between coherence time $T_{C}$ and speed was calculated. Then, this was compared with the actual symbol duration $t_{s}$ for a LoRaWAN transmission, taking into account the different SFs, which impact the actual $t_{s}$, as discussed in Section 3.

**Figure 3:**
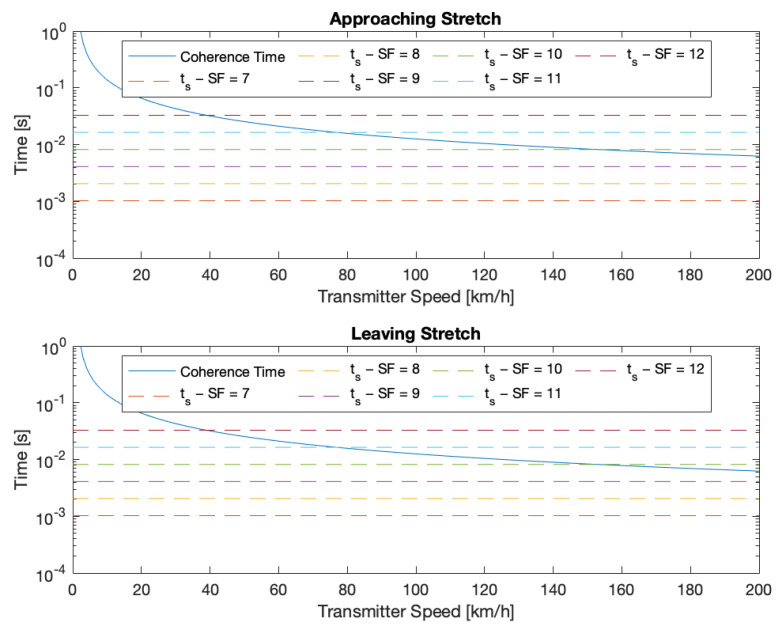
Comparison between symbol time $t_{s}$ for each SF and coherence time $T_{C}$ in function of the speed.

**Figure 4:**
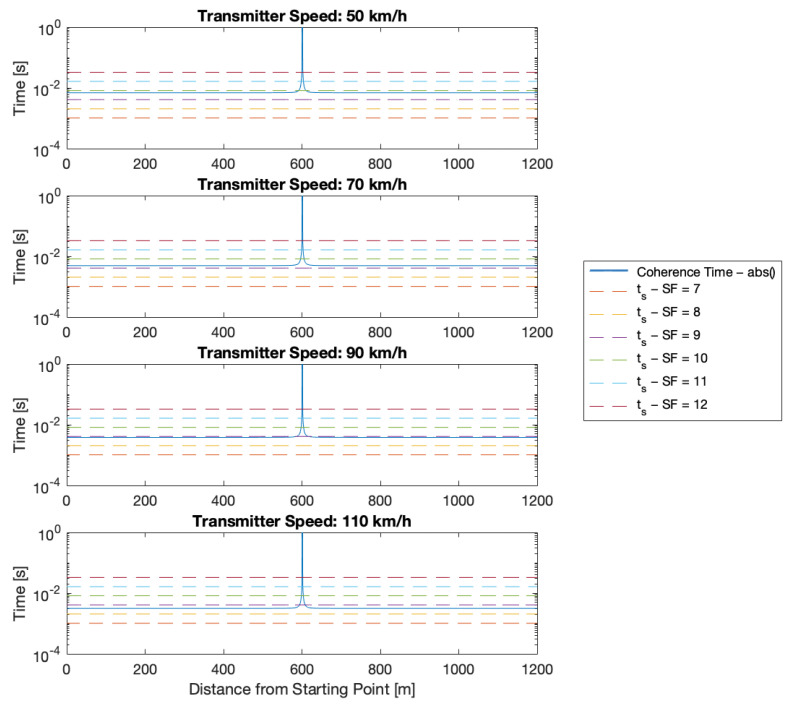
Comparison between symbol time $t_{s}$ for each SF and coherence time $T_{C}$ for each tested speed.

In particular, in Figure 3, the $T_{C}$ trend is compared with the $t_{s}$ achievable for the six SFs, while Figure 4 focuses explicitly on the six speeds at which the tests were carried out. Concerning this last figure, the relative speed between the transmitter and the gateway has to be taken into account. While this speed can be considered constant when the transmitter is far from the gateway, it rapidly decreases to 0km/h and then increases again when the transmitter transmits in front of the gateway. At this point, the car switches from approaching to leaving the gateway; this is the reason for the peak of the coherence time in the middle of the path.

Looking at the two figures, one can see that, resorting only to the theory, successful transmission is expected to be possible when in motion only for SFs from 7 to 9 and for the two lowest speeds (i.e., at 50km/h and 70km/h). At 90km/h, the transmission is expected to also become problematic for SF 9, while at 110km/h, the transmission at this SF appears to be totally unfeasible.

## 6. Results

### 6.1. Velodrome Measurement Campaign

**Figure 5:**
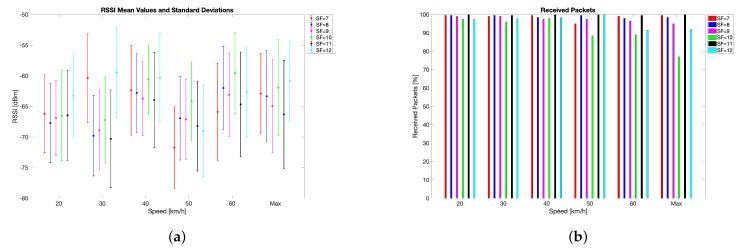
Test results related to the velodrome measurement campaign’s quantitative analysis: (**a**) RSSI and (**b**) received packets (picture taken from [11]).

**Figure 6:**
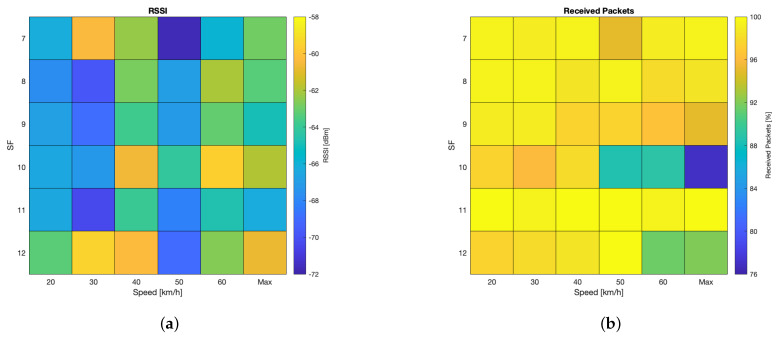
Test results related to the velodrome measurement campaign’s qualitative analysis: (**a**) RSSI and (**b**) received packets (picture taken from [11]).

The test results related to the velodrome measurement campaign are reported in Figure 5 and Figure 6. First of all, the robustness of the LoRa modulation, and therefore of the LoRaWAN protocol, was confirmed. This hints at the potential feasibility of a vehicular network relying on such technologies, provided that low latency and high QoS are not requisites of utmost importance for the application at hand, since it is well known that the LoRaWAN protocol is lacking in these regards. The analysis of RSSIs highlighted that no macroscopic correlation with speed can be observed. However, RSSIs varied throughout the tests, although they did not hinder the wireless link. Particularly, the mean RSSIs spanned from −59 dBm to −72 dBm, meaning that a considerable link margin was experienced. Indeed, the LoRaWAN gateway had a sensitivity ranging from −137dBm at SF = 12 to −126dBm at SF = 7 (which are common values for many other commercial LoRaWAN gateways). Moreover, the measured RSSI excursion of 13dB is almost a tenth of the receiver sensitivity. Finally, the technology’s robustness was also validated by the limited values of RSSI standard deviations, which were approximately constant during the tests forming the measurement campaign.

The percentage of received packets was almost always 100% apart from a few minima: 97.5% at 20km/h and 40km/h, 96.0% at 30km/h, 88.5% at 50km/h, and 89.0% at 60km/h; the overall minimum (i.e., 77.0%) was recorded during the ’Max’ test set. Nonetheless, such results are far from being unexpected since a PL spanning from 20% to 30% should be taken into account in applications involving radio technologies operating in unlicensed frequencies (e.g., LoRa).

Finally, the test results can be analysed from the point of view of the SF. According to the tests, a worst-case scenario can be identified for SF = 10 because transmission performances marginally deteriorate as speed increases. However, owing to the limited number of transmitted packets (i.e., 200 per SF per speed), such a result can likely be ascribed to statistical fluctuations rather than to movement.

### 6.2. Straight Road Measurement Campaign

**Figure 7:**
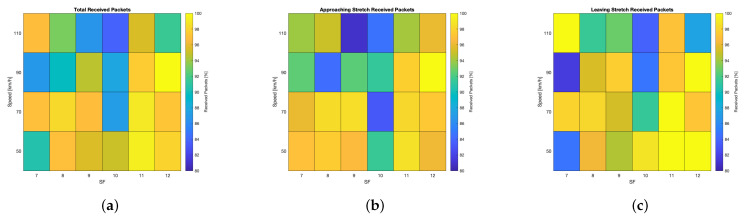
Test results related to the straight road measurement campaign’s qualitative analysis of the received packets: (**a**) total; (**b**) approaching stretch; (**c**) leaving stretch.

**Figure 8:**
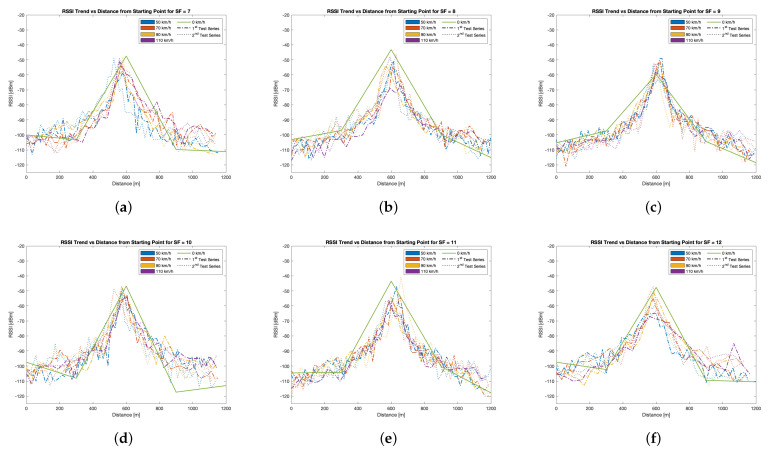
Test results related to the straight road measurement campaign’s quantitative analysis of the RSSIs: (**a**) SF = 7; (**b**) SF = 8; (**c**) SF = 9; (**d**) SF = 10; (**e**) SF = 11; (**f**) SF = 12.

The test results related to the straight road measurement campaign are shown in Figure 7 and Figure 8. Similarly to the other set of tests, the robustness and feasibility of LoRa modulation in this application scenario were proven. However, such viability may fail if the specific application scenario cannot tolerate high latency and low QoS because the LoRaWAN protocol implicitly entails such characteristics. Since the distance covered by the uplinks varied throughout the tests, the relative results are analysed by considering two stretches: the approaching one, meaning that the transmitter was getting closer to the gateway, and the leaving one, during which it was getting further away. This distinction helps in assessing probable effects due to Doppler.

Firstly, let us take into account the performance from the perspective of the received packets. On the whole, Figure 7a shows that, apart from a few cases in which the received packets ranged from 84% to 88%, the bulk of the tested settings recorded a percentage of received packets ranging from 90% to 100%, underlining the robustness of LoRaWAN for this application. Specifically, for speeds greater than or equal to 90km/h, the PL increased, as was suggested in the literature. However, contrary to related works, adopting higher SFs ensured better reception capabilities, although the symbol time increased. This is justified by the fact that LoRa modulation is intrinsically robust against the Doppler effect (see Section 5.2), thus making it eligible for V2R communications. This is also confirmed by analysing Figure 7b,c: if compared, no significant discrepancies arise between what concerns the approaching and the leaving stretches and what concerns the percentage of received packets; moreover, these results are consistent with the results of Figure 7a, meaning that the transmission architecture worked similarly in both stretches.

Secondly, let us take into account the performance from the point of view of the RSSI measurements. Such results are displayed for each of the tested SFs, and they are compared with the set of RSSI measurements performed by keeping the transmitter still at the fixed points, thus allowing us to be able to assess the probable effects of movement and of the Doppler effect. In each of the charts of Figure 8, such measurements are plotted with a solid green line, while the RSSIs related to the tests accounting for movement are represented with dashed and dotted lines, with the colour representing the tested speed. Since the wireless links had variable distances, evaluating the RSSIs by looking for the best values is not meaningful (i.e., it is trivial that the best RSSIs were observed at halfway, close to where the gateway was installed). Conversely, the aim of this test is to spot whether, for a given SF, movement played a significant detrimental role, and to evaluate whether the RSSIs significantly varied for each of the SFs. Tests related to SF = 7 (see Figure 8a) showed that RSSIs varied, regardless of the speed, with respect to those recorded with no motion. Specifically, some of them were slightly worse with respect to those at 0km/h, while some others were slightly better. This indicates that no significant variations can be perceived, and that a minimal link margin was experienced since the receiver sensitivity at SF = 7 is −126dBm (which is the worst achievable for the tested transmitting parameters). The same conclusion can be drawn for the measurements performed at SFs of 8, 9, and 11. But, for those SFs, better receiver sensitivity was available, meaning that larger link margins were present. On the other hand, measurements related to SFs of 10 and 12 exhibited a slightly different behaviour, since the RSSIs recorded when the transmitter was still were higher for some tested spots with respect to those of the other SFs. However, such an outcome can be due to exogenous factors due to environmental conditions that cannot be controlled since tests were performed outside a laboratory, and it does not imply that movement played a significant beneficial role in the wireless links. Therefore, on the whole and on the face of such tests, it can be concluded that movement and the Doppler effect had a limited effect on RSSIs and a marginal effect on PL when low SFs were exploited and high speeds were tested. We are aware that several speeds could have been tested, but testing lower speeds would have modelled better-case scenarios, while testing higher ones would have been dangerous owing to the morphology of the straight road. Moreover, and at least in Italy, the maximum speed limit is 130km/h, which can be achieved in motorways only.

## 7. Discussion

The most promising result that emerged from the two measurement campaigns is that LoRa modulation and the LoRaWAN protocol can potentially enable vehicular networks, at least for the V2R paradigm. Indeed, PL, and in general performance decrease, was in line with the intrinsic behaviour of the technology, where operating in ISM bands is inevitably subject to interference and noise. Thus, if the application scenario allows a minimal amount of data loss along with a negligible latency, LoRaWAN can be a valuable alternative.

During the first measurement campaign, a quantitative analysis of the RSSI was possible since the length of the wireless path was almost constant throughout all the experiments. Conversely, RSSIs of the second measurement campaign could only be examined from a qualitative point of view since the path length varied over the trials. Indeed, just a comparison with the RSSI measurements performed with the transmitter kept still could be made. However, tests on RSSIs showed, on the one hand, that minimal variation was experienced when the speed changed, which did not mind the effectiveness of LoRa owing to its robustness, and on the other hand, that such measurements marginally varied for a given spot when the transmitter was moved with respect to when it was still. Both of these outcomes were independent of the SF, since for the velocity at hand, minor changes were experienced for different SFs. Of course, a deep analysis of link margin is superfluous and not pertinent because, in both measurement campaigns, the path length was particularly limited: in the first one, from a minimum of 35.0m to a maximum of 67.5m, and in the second one, from 6m to 600m.

The other metric under investigation was the PL or, alternatively, the percentage of received packets. For the measurement campaign conducted in the velodrome, the PL was almost always negligible, apart from five testing setups. For all of the other settings, the percentage of received packets ranged from 96% to 100%. A similar behaviour was observed also in the tests held on the straight road, although the number of testing setups registering a percentage of received packets below 96% was more consistent, but in the worst case, 84% of transmitted packets were received, meaning that the technology can be considered robust nonetheless since it performed according to what other related works dealing with LoRa links in harsh contexts reported. Moreover, for the latter campaign, the PL was analysed by considering the approaching and leaving stretches in addition to the overall performance. This was performed in order to assess probable detrimental effects due to the Doppler effect. However, since the results related to the three analyses (i.e., overall, the approaching stretch, and the leaving stretch) were similar, it can be concluded that the Doppler effect had a limited, almost negligible effect. Nevertheless, an additional fact must also be stressed. Since a gateway was exploited as the receiver rather than a spectrum analyser, it is reasonable that many of the lost packets could have been received, but they could have been corrupted, thus being marked as lost by the gateway due to its inability to demodulate them. Contrarily, if a spectrum analyser was exploited, then the PL would be far lower. This would have been a best-case scenario apart from the fact that, in real-life applications, gateways are almost always employed in place of spectrum analysers, and that the ability to retrieve information coded within the packet payloads is what is actually needed to consider the application satisfactory and effective. The usage of a spectrum analyser would have been beneficial to assess whether some potential interference was present during experiments. However, since the tests for both measurement campaigns took place over a limited timespan (i.e., a few hours), if some interference affected the communication channel, thus acting as background noise, this would have been experienced over all of the RSSI measurements, hence making it negligible when such results are analysed from a relative perspective, as was proposed above. In light of this, we opted for the usage of just a gateway, thus testing at the same time a worst-case scenario (also because the maximum CR was selected) and a real-life application scenario.

Concerning the Doppler effect, from Figure 3 and Figure 4, one can see that the coherence time related to the Doppler effect is shorter than the symbol time, but the performance in terms of packet delivery does not degrade. This seems like a surprising result, but, in fact, it is not. The explanation of this result comes from the fact that Semtech’s LoRa receivers that are employed in the experiments can cope with a considerable Doppler shift.

The technical possibility of a LoRaWAN receiver of coping with Doppler shifts of up to BW/4 is confirmed in the scientific literature. Quoting from [31]:

The LoRa receiver studied in this work can only estimate a CFO $\Delta f_{c}\in \left[-(B/4),(B/4)\right]$, where CFO stands for Carrier Frequency Offset (equivalent in this context to the Doppler shift) and *B* denotes the bandwidth BW.

In Section 2.1 of [32], an application note covering Semtech’s components used in the experiments, it is first of all confirmed that the maximum allowed Doppler shift is 1/4 of the bandwidth. A budget of the residual allowed frequency shift resulting after the consideration of an error of 5ppm for the gateway and an error of 25ppm for the transmitter is provided. In the case of BW=125kHz, the remaining frequency drift budget allowed is 5.21kHz; considering a centre frequency for the communication of 868MHz, a Doppler shift of 5.21kHz corresponds to a speed of 6482.52km/h or 5.3Mach. Of course, with a larger bandwidth, the limit increases. The tolerance of LoRa receivers to Doppler shifts is also confirmed by other related works in the literature, whose results support those of this paper, along with those of [20,21,22,23,26,27,28], which were extensively analysed in Section 2. For instance, [33] performed tests investigating the performance of off-the-shelf LoRa transceivers in real-life contexts (that match with the measurement campaigns of Section 5), finding comparable results with the ones of this paper, although only the maximum SF was tested. In the same vein, [34] also performed similar experiments with respect to those of Section 5.2 and obtained analogous results, although a different bandwidth was exploited for the uplinks. Therefore, it is reasonable to deem that the minimal receiving performance decay experienced in the straight road measurement campaign, where the RSSIs related to the moving transmitter were compared with the ones of the still transmitter, can be ascribed to other detrimental effects (e.g., multipath fading) rather than to the Doppler effect that typically hinder wireless communications enabled by radio frequency technologies like LoRa modulation. Moreover, such an effect becomes more prominent whenever unlicensed frequency bands, like ISM bands, are exploited.

We can conclude then that, thanks to the possibility of LoRaWAN receivers, even those that are commercially available such as those that have been used in the experiments, to tolerate high Doppler shifts, the results of our experiments are perfectly justified. However, these promising results do not compensate for the intrinsic limitations of LoRa modulation and the LoRaWAN protocol when applied to the context of vehicular networks. For instance, this may not be the best alternative when the application scenario requires URLLC or massive IoV, since the LoRaWAN protocol implicitly entails a non-negligible latency and a low QoS. In such cases, cellular technologies (e.g., 5G) are definitely better suited. But this also implies a rise in running costs since, conversely to LoRaWAN, data transmission plans must be subscribed to. Thus, during the design phase of the network enabling the application, a thorough trade-off has to be generally achieved bearing in mind all of the use case requirements.

## 8. Conclusions

This paper aimed at assessing the feasibility, effectiveness, robustness, and reliability of the LoRa modulation and of the LoRaWAN protocol when exploited for enabling vehicular networks, specifically for V2R paradigms. To this aim, two measurement campaigns were conducted: the former took place in a velodrome, by putting a LoRaWAN transmitter inside the tail trunk of a motorcycle and by installing a LoRaWAN gateway in the centre of the velodrome; the latter was performed on a straight road, by placing a transmitter in a car with the antenna on the car roof and by installing the gateway at the halfway point in a lateral position with respect to the road. Several speeds were examined, and all of the SFs were tested in order to validate as many configurations as possible. Moreover, during the second set of tests, the Doppler effect was evaluated. The test results proved the overall feasibility of the transmission system for the specific application scenario, highlighting a degradation in reception performance in terms of PL and RSSI as the speed increased that, however, did not undermine the effectiveness of the wireless communication technology. Moreover, during the latter testing campaign, it was found that the Doppler effect played a marginal detrimental role, thus remarking the robustness of the LoRa modulation in terms of the frequency tolerance of the receivers.

Future work will address the current limitations of this study. Tests accounting for multiple moving transmitters will be conducted, as well as those accounting for multiple gateways. This can potentially pave the way for further tests within a smart city scenario, in which a multitude of attenuation sources are present (e.g., buildings, trees, other vehicles, etc.). In so doing, a broader perspective could be obtained, thus extensively validating the results obtained so far. Another limitation of the proposed approach is that the aforementioned presented and discussed results derive from measurement campaigns conducted in a velodrome and on a straight road. Although such results can be fully exploited to provide readers and practitioners in the field with useful insight about the topic, they could not be potentially extended to further contexts, at least as a first instance, unless additional efforts or assumptions are made. Nonetheless, future work addressing multiple moving transmitters and multiple gateways will shed light on this.

## Data Availability

Data are contained within the article.
